# Lower ultra-short-term heart rate variability can predict worse mucosal healing in ulcerative colitis

**DOI:** 10.1186/s12876-023-02823-2

**Published:** 2023-05-29

**Authors:** Jianan Guo, Wenguo Chen, Huatuo Zhu, Hongtan Chen, Xiaodong Teng, Guoqiang Xu

**Affiliations:** 1 Department of Gastroenterology, The First Affiliated hospital, Zhejiang University School of Medicine, No. 79 Qingchun Road, Hangzhou, 310003 People’s Republic of China; 2 Department of Pathology, The First Affiliated hospital, Zhejiang University School of Medicine, No. 79 Qingchun Road, Hangzhou, 310003 People’s Republic of China

**Keywords:** Ulcerative Colitis, Heart Rate Variability, Psychological Stress, Mucosal Healing, Histological Healing

## Abstract

**Background:**

Psychological stress has been proved to be a risk factor for exacerbation for ulcerative colitis (UC). However, traditional approaches of quantifying psychological stress using psychological scales are time-consuming and the results may not be comparable among patients with different educational levels and cultural backgrounds. Alternatively, heart rate variability (HRV) is an indicator for psychological stress and not biased by educational and cultural backgrounds.

**Aims:**

In this study, we try to explore the relationship between psychological stress and UC by analyzing the effect of ultra-short-term HRV on mucosal and histological remission status of UC.

**Methods:**

This is a retrospective case–control study on UC inpatients from 2018 through 2020. Ultra-short-term HRV were calculated using baseline electrocardiography. Patients were divided intocase and control groups according to their Mayo endoscopic scores or histological Geboes scores. Three variables of ultra-short-term HRV (the standard deviation of normal to normal R-R intervals (SDNN), the standard deviation of successive differences between adjacent normal to normal R-R intervals (SDSD), the root mean square of successive differences of normal to normal R-R intervals (RMSSD)) were compared between different groups. And for those variables with significant differences, we built univariate and multivariate logistic regressions to depict the relationship between HRV variables and remission status of UC.

**Results:**

All three HRV variables showed significant differences between the mucosal groups. However, none of them showed significant difference between the histological groups. In further logistic regression analyses, smaller RMSSD can predict severe mucosal healing status (OR = 5.21).

**Conclusions:**

Lower ultra-short-term HRV (i.e. smaller RMSSD) is shown to positively correlate with worse mucosal healing status. However, ultra-short-term HRV cannot predict histological healing status according to our data.

## Introduction

Ulcerative colitis (UC) is a chronic disease with remitting and relapse that brings heavy burden both physically and psychologically to patients. Psychological stress is considered a risk factor for UC exacerbation. The association between heart rate variability (HRV) and psychological stress has been widely accepted [1]. Significant correlation has been observed between long-term HRV and stress in UC patients in a pilot study [2], while short-term HRV is also considered an objective biomarker for psychological stress both consciously recognized or subconsciously existed [3].

Ultra-short term HRV is calculated using data from an electrocardiograph (ECG) that is shorter than five minutes. Compared with long-term HRV, it is more feasible in daily clinical practice, with high reliability and acceptably low bias [4], especially when the ECG data is collected under static conditions [5]. Meanwhile, ultra-short-term HRV is shown to be sensitive in detecting psychological stress in real life [6, 7]. Based on those merits, ultra-short-term HRV is a reasonable biomarker for quantifying psychological stress among UC patients.

However, the relationship between the ultra-short-term HRV and the severity of UC has not been directly demonstrated yet. This study is designed to use ultra-short-term HRV as a metric to explore the relationship between psychological stress and the severity of UC. This is a retrospective case–control study using baseline ultra-short-term HRV as an objective measurement of psychological stress in UC patients. The case and control groups were naturally divided by their mucosal or histological inflammatory status. In this study we characterize the relationship between the baseline ultra-short-term HRV and the inflammatory status of UC patient, and develop a predictive model of mucosal or histological remission status of UC.

## Methods

In this retrospective case–control study, we analyzed the clinical data from patients with a primary diagnosis of ulcerative colitis and who were admitted in the First Affiliated Hospital of Zhejiang University between January 1^st^, 2018 and December 31^st^, 2020 (including both days). The diagnosis of UC in all participants included was confirmed by both colonoscopy and pathology of biopsy. Detailed inclusion and exclusion criteria are listed in Table 1. The two key criteria to evaluate the disease activity is the Mayo endoscopic score (MES) and the histological Geboes Score (GS), which reflect the baseline mucosal and the histological inflammatory status, respectively. Colonoscopy images before treatment adjustment were reviewed by a senior gastroenterologist and their MES was recorded. Histological images of colonic biopsy were reviewed by a senior pathologist and their GS was recorded. Patients were divided into different groups according to the MES and the GS. Specifically, in the evaluation of the mucosal inflammatory status, patients with a MES of 2 or 3 were defined as “severe”, while patients with a MES of 0 or 1 were defined as “mild-moderate”. In terms of the histological inflammatory status, a GS less than 2 was defined as mucosal healing, while a GS less than 3.1 was defined as mucosal remission. The baseline demographic information, the disease duration, the treatment adjustment, and the baseline inflammation status of the participants were also collected. Here the baseline inflammation status includes the white cell count, the C-reactive protein (CRP) and the erythrocyte sedimentation rate (ESR). The three variables used to evaluate the ultra-short-term HRV in this study include: the standard deviation of normal to normal R-R intervals (SDNN), the root mean square of successive differences of normal to normal R-R intervals (RMSSD), and the standard deviation of successive differences between adjacent normal to normal R-R intervals (SDSD). They were calculated based on the time-domain methods using the baseline 10-s electrocardiography at patient admission. All diagnosis of electrocardiography and the calculation of HRV was double-checked by a cardiologist.

**Table 1:** Inclusion and exclusion criteria

Inclusion criteria	Exclusion criteria
All admitted patients with the primary discharge diagnosis of ulcerative colitis	Complicated with any one of the conditions: sepsis, septic shock, active gastrointestinal bleeding, heart failure with NYHA Class II-IV, myocardial infarction or stroke within 3 months, liver cirrhosis with decompensation, chronic kidney disease stage IV or V Female patients who are pregnant
The diagnosis of ulcerative colitis is established by symptoms, colonoscopy and histology	ECG with any one of the conditions: non-sinus rhythm, ventricular pre-excitation syndrome, any atrial or ventricular premature beat on ECG recording, bradycardia with resting heart rate less than 50 bpm, tachycardia with resting heart rate more than 120 bpm
Have at least one colonoscopy with biopsy during admission	Taking any one type of the medications a week before or during admission: beta-blocker, muscarinic cholinergic-blocker/agonist
Have at least one standard 12-leads ECG during the first 24 h of admission	Active infection of any of the pathogens: mycobacterium tuberculosis, CMV, EBV, HAV, HBV, HCV, HDV, HEV

*Abbreviations:* *ECG* Electrocardiogram, *NYHA* New York Heart Association, *CMV* Cytomegalovirus, *EBV* Epstein-Barr virus, *HAV* Hepatitis A virus, *HBV* Hepatitis B virus, *HCV* Hepatitis C virus, *HDV* Hepatitis D virus, *HEV* Hepatitis E virus

### Statistics

The categorical variables in this study were analyzed by the Pearson 𝓧2 test while the quantitative variables were analyzed using Student’s t test. The association between HRV and severity of UC was evaluated via univariate and multivariable logistic regression models and odds ratio (OR) with 95% confidence intervals (CI). Univariate logistic regression was applied to detect possible association between the variables and the outcomes of interest. Multivariate logistic regression was applied to differentiate the real predictor out of potential confounders. All data were analyzed by the software SPSS (version 26.0, Chicago, IL, USA). A *p*-value less than 0.05 was considered statistically significant.

### Ethical approval

This study was approved by the Ethics Committee of the First Affiliated Hospital, Zhejiang University School of Medicine (Prot No 2022086).

## Results

### Baseline demographic information and inflammatory status

**Fig. 1:**
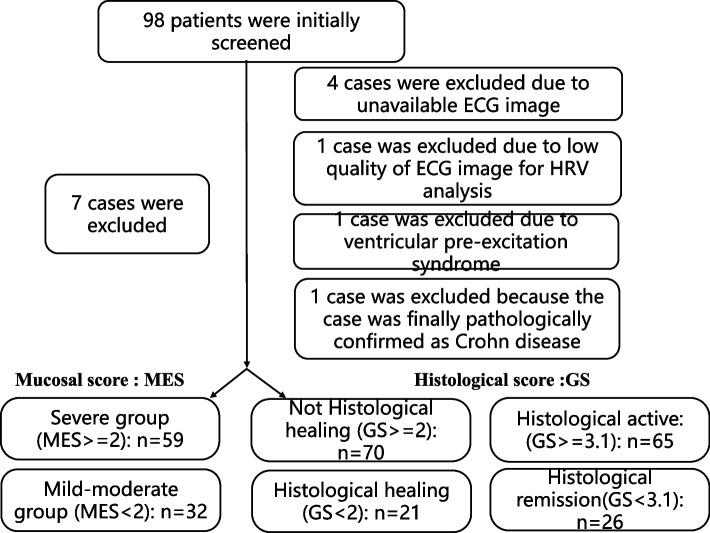
Patients screening and grouping

We finally included 91 patients in this study. Detailed information about the inclusion and exclusion criteria were listed in Fig. 1. The included patients had a mean age of 47.89 ± 13.44 years, a mean disease duration of 4.64 ± 6.23 years, and a mean hospital stay length of 11.94 ± 10.77 days. Based on the MES, 59 patients (with MES 2 or 3) were assigned to the “Severe group” while 32patients (with MES 0 or 1) were assigned to the “Mild-Moderate group”. Similarly, based on the Geboes Scores (GS) calculated from their histological images, 70 patients with GS ≥ 2 were considered not achieve histological healing while 21 patients with GS < 2 were considered histological healed. When an alternative GS cut-off of 3.1 was used, 65 patients (with GS ≥ 3.1) were considered histological active while the other 26 patients (with GS < 3.1) were considered histological inactive.

Baseline demographic distributions and basic disease characteristics (including age, gender, disease duration, and therapy categories) and D-dimer level analysis did not show statistically different between the two MES groups. Fecal calprotectin level was not statistically different between the two MES groups either. However, it is noteworthy that fecal calprotectin was not commonly tested in 2018 in this hospital, and the lack of this data could lead to bias in the results. Detailed information is listed in Table 2.

**Table 2:** Baseline demographic information and disease characteristics, inflammation status, therapy distribution according to mucosal healing status

	**Severe group (*N* = 59)**	**Mild-Moderate group (*N* = 32)**	***P*-Value**
Age, year, mean (SD)	47.02 (13.11)	49.50 (14.10)	0.40
Male, n (%)	37 (62.71)	18 (56.25)	0.547
Disease duration of UC, years (SD)	4.19 (4.97)	5.47 (8.08)	0.42
Newly diagnosed cases (%)	9 (15.25)	2 (6.25)	0.208
Hospital stay length, days (SD)	15.58 (11.58)	5.25 (3.88)	** < 0.001**
Frequency of bowel movements, times/day (SD)	6.66 (3.86)	2.16 (1.68)	** < 0.001**
Hematochezia, n (%)	55 (93.22)	12 (37.5)	** < 0.001**
WBC count, *10^9/L, (SD)	8.01 (3.02)	6.05 (2.20)	** < 0.001**
Hemoglobin, g/L, (SD)	109.14 (24.80)	135.16 (19.17)	** < 0.001**
Hct, %, (SD)	33.98 (6.85)	40.97 (4.74)	** < 0.001**
Platelet count, *10^9/L, (SD)	327.17 (110.98)	229.31 (63,07)	** < 0.001**
C-reactive protein, mg/L, (SD)	45.04 (51.89)	3.08 (6.37)	** < 0.001**
ESR, mm/h, (SD)	27.15 (21.44)	7.53 (5.83)	** < 0.001**
D-dimer, µg/L, (SD)	1740.27 (3724.00)	798.20 (2019.04)	0.189
Fecal Calprotectin (μg/g)	N = 36	N = 15	
	323.88 (285.57)	195.55 (315.50)	0.162
Albumin, g/L, (SD)	34.95 (5.65)	42.25 (5.10)	** < 0.001**
Current therapy by 5-ASA (%)	49 (83.05)	28 (87.5)	0.797
Current corticosteroids drugs (%)	8 (13.56)	3 (9.38)	0.804
Current anti-TNF treatment (%)	1 (1.69)	0 (0)	1.000

*Abbreviations:* *SD* Standard Deviation, *ESR* Erythrocyte sedimentation rate, *ASA* Aminosalicyclic acid, TNF Tumor necrosis factor

According to histological remission using GS score 2 as a cut-off point, age, gender distribution, disease duration, therapy distribution and D-dimer level were not statistically different between the two groups. These were consistent to endoscopic remission evaluation using MES. However, when switch the cut-off point of GS score to 3.1 in differentiate histological active and inactive, variate differences were similar except that D-dimer was statistically different between the two groups. Detailed information is listed in Table 3.

**Table 3:** Baseline demographic information and disease characteristics, inflammation status, therapy distribution according to histological healing status

	**Not histological healing** ***N* = 70 (GS > = 2)**	**Histological healing** ***N* = 21 (GS < 2)**	***P*-Value**	**Histological active** ***N* = 65 (GS > = 3.1)**	**Histological inactive** ***N* = 26 (GS < 3.1)**	***P*-Value**
Age, year, mean (SD)	46.71 (14.71)	51.81 (9.72)	0.067	46.82 (14.45)	50.58 (10.25)	0.230
Gender (male, %)	43 (61.43)	12 (57.14)	0.725	41 (63.08)	14 (53.85)	0.416
Hematochezia (%)	60 (85.71)	7 (33.33)	** < 0.001**	55 (84.62)	12 (42.86)	** < 0.001**
Disease duration of UC, years (SD)	3.70 (4.15)	7.80 (10.07)	0.082	3.64 (4.11)	7.15 (9.34)	0.076
Newly diagnosed cases (%)	10 (14.29)	1 (5.00)	0.240	8 (12.31)	3 (11.54)	0.919
Hospital stay length, days (SD)	13.55 (11.31)	6.57 (6.46)	**0.008**	13.89 (11.64)	7.07 (6.09)	**0.006**
WBC, *10^9/L, (SD)	7.86 (3.01)	5.55 (1.54)	** < 0.001**	8.02 (2.99)	5.60 (1.78)	** < 0.001**
Hemoglobin, g/L, (SD)	113.66 (26.63)	133.71 (16.88)	** < 0.001**	113.69 (26.79)	129.77 (20.37)	**0.007**
Hct, %, (SD)	35.20 (7.22)	40.58 (4.28)	** < 0.001**	35.22 (7.34)	39.48 (5.06)	**0.002**
Platelet count, *10^9/L, (SD)	313.23 (110.52)	224.52 (57.63)	** < 0.001**	320.80 (111.08)	222.65 (52.09)	** < 0.001**
C-reactive protein, mg/L, (SD)	38.02 (50.26)	4.51 (8.67)	** < 0.001**	40.80 (51.12)	4.00 (7.88)	** < 0.001**
ESR, mm/h, (SD)	23.76 (21.28)	8.57 (6.08)	** < 0.001**	24.33 (21.76)	10.04 (8.01)	** < 0.001**
D-dimer, µg/L, (SD)	1658.13 (3661.47)	578.53 (623.96)	0.184	1752.25 (3784.29)	550.85 (576.66)	**0.015**
Albumin, g/L, (SD)	36.48 (6.21)	41.00 (6.22)	**0.004**	36.20 (6.28)	40.81 (5.80)	**0.002**
Fecal Calprotectin (μg/g)	*N* = 43	*N* = 7		*N* = 41	*N* = 9	
	273.08 (268.56)	294.21 (434.10)	0.861	310.69 (304.19)	118.19 (153.20)	0.072
Current therapy by 5-ASA (%)	59 (84.29)	18 (85.71)	0.874	56 (86.15)	21 (80.77)	0.520
Current corticosteroids drugs (%)	10 (14.29)	1 (4.76)	0.240	9 (13.85)	2 (7.69)	0.416
Current anti-TNF treatment (%)	1 (1.43)	0 (0)	0.582	1 (1.54)	0 (0)	0.525

*Abbreviations: SD* Standard Deviation, *ESR* Erythrocyte sedimentation rate, *ASA* Aminosalicyclic acid, *TNF *Tumor necrosis factor

### The relationship between ECG-based ultra-short-term HRV and mucosal and histological remission status

Ultra-short-term HRV was calculated based on each patient’s 10-s-ECG in the first 24 h upon admission using time-domain analysis. HRV variables including SDNN, SDSD, RMSSD and the heart rate (HR) were compared among different groups. All HRV variables showed significant differences between the “Severe group” and the “Mild-Moderate group” based on MES. The “Severe group” has lower SDNN, SDSD and RMSSD and higher HR compared with the “Mild-Moderate group”. However, when evaluating the histological status using GS, no HRV variables showed significant difference either between groups with a GS cut-off of 2 nor 3.1. Detailed information is listed in Table 4.

**Table 4:** HRV analysis

HRV	MES > = 2 *N* = 59	MES < 2 *N* = 32	*P*-Value	GS > = 2 *N* = 70	GS < 2 *N* = 21	*P*-Value	GS > = 3.1 *N* = 65	GS < 3.1 *N* = 26	*P*-Value
SDNN (ms, SD)	18.54 (9.15)	29.33 (25.00)	**0.024**	20.75 (17.81)	27.61 (14.15)	0.11	20.86 (18.40)	26.00 (13.40)	0.20
SDSD (ms, SD)	11.72 (6.23)	19.95 (20.96)	**0.037**	13.65 (13.66)	17.82 (14.38)	0.21	13.63 (14.08)	17.05 (13.03)	0.29
RMSSD (ms, SD)	12.31(6.61)	21.42 (22.23)	**0.030**	14.37 (14.50)	19.33 (15.28)	0.18	14.35 (14.94)	18.43 (14.09)	0.24
HR (bpm, SD)	78.59 (12.91)	72.88 (13.01)	**0.047**	77.79 (12.86)	72.57 (13.67)	0.11	78.12 (18.87)	72.73 (13.34)	0.08

*Abbreviations: SDNN* The standard deviation of normal to normal R-R intervals, *SDSD* The standard deviation of successive differences between adjacent normal to normal R-R intervals, *RMSSD *The root mean square of successive differences of normal to normal R-R intervals

Receiver operating curves (ROC) were made to decide the optimal cut-off to transfer quantitative variables into categorical. According to the calculated maximum Youden index, the cut-offs for SDNN, SDSD and RMSSD should be 16.807 ms, 14.721 ms, 15.516 ms and 77.5beats/min, respectively. Cut-offs for other quantitative variables were decided using either commonly used or previously published clinical references [8, 9].

There is a linearity between all independent variables and log-odds using Box-Tidwell test. Absence of multi-collinearity among independent variables is confirmed using linear regression. The tolerance of all variables is larger than 0.1 and the variance inflation factors (VIF) are all less than 10, indicating that there is no multi- collinearity among independent variables. This justifies the use of logistic regression on our data. The accuracy, sensitivity and specificity for our model was 89.0%, 84.4% and 91.5%, respectively. Our logistic regression model's goodness of fit in the Hosmer and Lemeshow Test is fair (*p* = 0.986), indicating that the regression model is trustworthy. We have included all significant variables under MES group into univariate analysis. Finally, RMSSD, hemoglobulin, CRP and ESR were variables screened by multivariate logistic regression analysis. Detailed information is listed in Table 5. Despite different distributions were observed among all of the HRV parameters include SDNN, SDSD and RMSSD between severe and mild-moderate UC patients, multivariate regression analysis included only lower RMSSD (RMSSD < 15.516 ms) is positively related to severe mucosal inflammation of UC (OR = 5.21).

**Table 5:** Univariate and multivariate logistic regression analysis

	**Univariate analysis**		**Multivariate analysis**	
Variable	**OR (95%CI)**	***P* value**	**OR (95%CI)**	***P* value**
SDNN < 16.807 ms	3.21 (1.29–7.97)	0.012		0.613
SDSD < 14.721 ms	3.64 (1.46–9.12)	0.006		0.635
RMSSD < 15.516 ms	5.36 (2.11–13.63)	< 0.001	5.21 (1.12–24.14)	**0.035**
Hb < 100 g/L	17.13 (2.18–134.60)	0.007	24.69 (1.84–330.40)	**0.015**
CRP > 5 mg/L	31.07 (8.21–117.65)	< 0.001	35.43 (6.91–181.63)	** < 0.001**
ESR > 20 mm/h	34.32 (4.39–268.17)	0.001	15.73 (1.34–184.54)	**0.028**
WBC > 10 × 10^9^/L	9.64 (1.21–77.18)	0.033		0.694
Hct < 38%	6.31 (2.43–16.40)	< 0.001		0.743
Platelet > 296 × 10^9^/L	10.70 (2.94–39.03)	< 0.001		0.161
Albumin < 35 g/L	11.82 (2.58–54.08)	0.001		0.503

## Discussion

### Ultra-short-term HRV can predict mucosal remission status

In this retrospective case–control study, we try to explore the potential relationship between ultra-short-term HRV and the severity of UC. We use both mucosal and histological scores to evaluate UC severity.

Significant differences in SDNN, SDSD and RMSSD were observed between the mucosal “severe” and “mild-moderate” groups. In further univariate and multivariate logistic regression model, lower RMSSD was detected as positively related with worse mucosal healing status (OR = 5.21). These results provided evidence that lower ultra-short-term HRV may be positively related to mucosal flare in UC. Notably, these results indicate that RMSSD may be used to predict the mucosal severity in UC, which provides a potentially non-invasive method for UC mucosal healing surveillance or even suggests new target for UC treatment.

### Discrepancy in that ultra-short-term HRV predicts mucosal remission but not histological remission

Since psychological stress is a risk factor for UC exacerbation, we have hypothesized that lower ultra-short-term HRV can predict for poorer mucosal healing status, however, we did not observe significant differences between different histological groups in our study, which suggests that ultra-short-term HRV is not a useful metric to predict the histological severity or remission status of UC. Therefore, our results indicate a discrepancy in that ultra-short-term HRV can predict the mucosal remission but not the histological remission.

One possible explanation for this discrepancy is the latency between histological healing and mucosal healing. Endoscopically quiescent UC may still be histological active according to a previous clinical study [10]. Patients with a MES of 0 may still have a high risk of relapse if they have histological basal plasmacytosis [11]. Our rationale is that during mucosal healing, patients may realize the improvement of the symptoms and this subjective cognition of improvement may bring positive feedback on their HRV. On the other hand, patients cannot “feel” the histological remission and therefore it doesn’t directly correlate with the HRV.

Though histological remission is widely accepted as a sensitive way in evaluating UC, the clinical application of histological healing as a treatment target is still controversial [12] and no clear criterion for histological remission has been consensually defined or validated. Moreover, in a prospective multi-center cohort study, the correlation between UC mucosal healing and histological healing is low [13]. These previous studies suggest that it is probably not necessary or feasible to expect consistent prediction of the two healing standards in one clinical model, which mitigates the concerns of the discrepancy observed in our study.

Lower ultra-short-term HRV, a variation of autonomic nerve tone, was found to be positively related to poor mucosal healing in ulcerative colitis patients in this study. Autonomic nerve system has a crucial role in modulating the relationship between stress and inflammation. Sympathetic nerve tend to be pro-inflammatory and parasympathetic tone has been proved to have potential role of anti-inflammatory effects in UC [14]. Moreover, vagal nerve stimulation is a promising new approach for UC control in a pilot study [15]. Among all three parameters involved in our study, RMSSD value correlated more to vagus nerve mediated heart activity [16]. It is interesting to explore the mechanism of this phenomenon One possible mechanism is that stimulation of vagal afferent fiber will have a systemic anti-inflammatory effect through splanchnic pathway [17]. Our work provides insight for non-invasive evaluation for the mucosal remission status of UC. Furthermore, bioelectronic medicine treatment like vagal nerve stimulation is considered has therapeutic potential in UC treatment [18]. These findings may give support for future treatment like vagal nerve stimulation.

The study we conducted had a few of limitations. It would be more reasonable if we design this study prospectively. Future prospective study focusing on UC flare may include quantification measurement of anxiety, depression and even life quality. We are convinced that it will offer greater evidence of the link between psychological stress and UC flare. Some subjective questionnaires to quantify the status of anxiety and depression may bring a multi-dimensional exploration of psychological stress and UC flare. Due to the limitation of retrospective observational study with limited sample size, a prospective clinical trial with larger sample size exploring the relationship of UC flare and psychological stress may provide more evidence.

## Data Availability

The datasets used in the current study are available from the corresponding author on reasonable request.

## Abbreviations

HRV: Heart rate variability
UC: Ulcerative Colitis
MES: Mayo endoscopic score
SDNN: The standard deviation of normal to normal
SDSD: The standard deviation of successive differences
RMSSD: The root mean square of successive differences
HR: Heart rate
GS: Geboes Score
ECG: Electrocardiograph
CRP: The C-reactive protein
ESR: Erythrocyte sedimentation rate
OR: Odds ratio
CI: Confidence intervals
